# Point Process Models for the Spread of Coccidioidomycosis in California

**DOI:** 10.3390/idr13020052

**Published:** 2021-06-16

**Authors:** Jiajia Wang, Ryan J. Harrigan, Frederic P. Schoenberg

**Affiliations:** 1 Department of Statistics, University of California, Los Angeles, CA 92521, USA; aaannie@ucla.edu; 2 Center for Tropical Research, Institute of the Environment and Sustainability, University of California, Los Angeles, CA 92521, USA; iluvsa@ucla.edu

**Keywords:** point process models, Coccidiodomycosis, valley fever, forecasting

## Abstract

Coccidioidomycosis is an infectious disease of humans and other mammals that has seen a recent increase in occurrence in the southwestern United States, particularly in California. A rise in cases and risk to public health can serve as the impetus to apply newly developed methods that can quickly and accurately predict future caseloads. The recursive and Hawkes point process models with various triggering functions were fit to the data and their goodness of fit evaluated and compared. Although the point process models were largely similar in their fit to the data, the recursive point process model offered a slightly superior fit. We explored forecasting the spread of coccidioidomycosis in California from December 2002 to December 2017 using this recursive model, and we separated the training and testing portions of the data and achieved a root mean squared error of just 3.62 cases/week.

## 1. Introduction

Coccidioidomycosis, known also as Valley fever, is a fungal mammalian disease caused by one of two saprophytic fungus species found in the desert Southwest of the United States and Mexico. Based on the epidemiological profile of coccidioidomycosis from the San Luis Obispo County Public Health Department [1], nearly 60% of people infected with coccidioidomycosis have minimal to no symptoms, while the remaining 40% experience a range of possible clinical symptoms including pneumonia, fatigue, cough, chest pain, and fever, and often require hospitalization. In a small percentage of cases, the disease disseminates to other parts of the body, including the skin, meninges, soft tissues, and bones, and can result in death.

In the United States, approximately 150,000 infections occur annually [2]. Although coccidioidomycosis has a higher annually cumulative total number of illness and death than many other diseases, coccidioidomycosis has attracted comparatively little attention over the past decade [3]. This lack of attention is somewhat surprising, given the disease’s relatively simple transmission cycle and presentation (a community-acquired pneumonia that is rarely transmitted between hosts) in a developed part of the world. Despite this, it is clear that more research and public health awareness are needed to understand the recent increase and spread of coccidioidomycosis (California incidence has risen 213% from 2014 to 2017 [4]) and to explore the role of statistical models in forecasting this spread both during and after an outbreak.

The self-exciting Hawkes point process model [5] has been commonly used to describe clustered phenomena, including earthquakes [6,7], crimes [8], invasive species [9], financial transactions [10,11], neuron activity [12], terrorist attacks [13], and contagious diseases [14]. The model is specified by a conditional rate λ(t) satisfying:

(1) $$\lambda \left(t\right)=\mu \left(t\right)+K\int_{0}^{t}g\left(t-t^{\prime }\right)dN_{t^{\prime }},$$

where μ(t) is the background rate, the parameter *K* is called the productivity, and the density function *g* is called the triggering function. Schoenberg et al. [15] argued that in modeling an epidemic, the expected number of transmissions for a subject infected at time *t* may depend on the conditional rate at time *t*. For instance, early in the outbreak of a disease, when the prevalence (or conditional rate) of the disease is low, the rate of transmission may be much higher than at later times when the virus has already spread, due to increased awareness, human mitigation efforts, and prior exposure to the disease. Schoenberg et al. [15] thus introduced the recursive model, which allows for changes in the productivity over time as the rate of incidence varies according to:

(2) $$\lambda \left(t\right)=\mu \left(t\right)+\int_{0}^{t}H\left(\lambda \left(t^{\prime }\right)\right)g\left(t-t^{\prime }\right)dN_{t^{\prime }}.$$

For the recursive model, the conditional intensity depends on *H*, which determines how the productivity varies with the conditional rate λ. Schoenberg et al. [15] proposed fitting $H\left(y\right)=\alpha y^{-q}$, showing improved fit for the recursive model compared to that of the simple Hawkes model in describing known cases of Rocky Mountain Spotty Fever in California between 1960 and 2011.

Park et al. [16] fit a Hawkes model for the spread of Ebola during the 2014 epidemic in Africa and showed that it described the spread of the virus better than the traditional SEIR model shown to fit well to the same data by Althaus [17]. Of particular interest here is whether the recursive model can outperform the Hawkes and SEIR models in its ability to fit epidemic data and to forecast future cases. We compared the models using 17 years of reported cases of coccidioidomycosis in California, from December 2002 to December 2017. Such a comparison may be useful not only for studying the spread of coccidioidomycosis, but also more generally as a test of the breadth of application of these types of models, applied successfully in the case of Ebola and plague, to a different class of communicable diseases with a very different transmission and infection cycle.

The structure of this paper is as follows. Following a description of the coccidioidomycosis data in Section 2, we briefly review the point processes models, recursive models, and methods of evaluation in Section 3. Our results are presented in Section 4, and Section 5 contains some concluding remarks.

## 2. Dataset

We used the coccidioidomycosis dataset [18] from Project Tycho, a database providing open access to U.S. notifiable disease data that have been reported by cities and states. The dataset contains information from external sources of disease surveillance data, such as the United States Centers for Disease Control or the World Health Organization.

**Figure 1:**
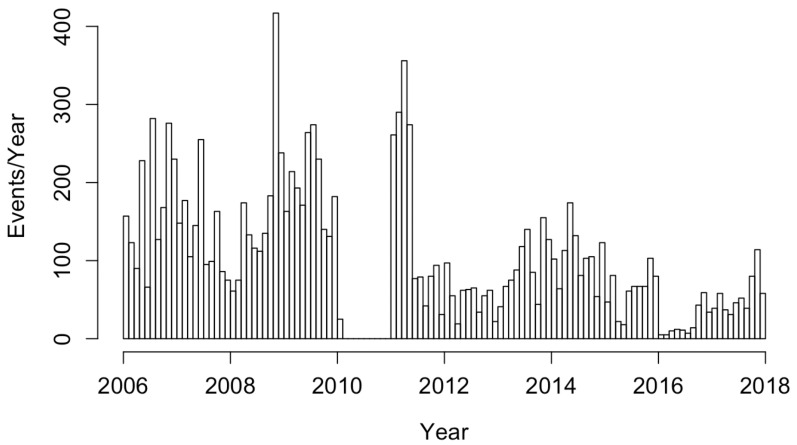
Histogram of coccidioidomycosis cases in California from January 2006 to December 2017.

Data on 12,202 reported case counts of coccidioidomycosis in California from 1 January 2006 to 16 December 2017 were obtained by the United States Centers for Disease Control and the World Health Organization and compiled by Project Tycho [18]. The dataset consisted of weekly statewide case totals during this time period. Weeks with no data over this time frame were treated as having no confirmed cases. When fitting point process models to the data, the estimated onset time for each infection within a given week period was randomly drawn from a uniform distribution covering the 7-day time interval as in Park et al. [16] and Schoenberg et al. [15]. No cases were reported between 3 January 2010 and 1 January 2011, as seen in Figure 1. We considered here data cataloged by Project Tycho, which included only the cases from the Morbidity and Mortality Weekly Report (MMWR), not annual data, which may have corrections or modifications made at year’s end, showing a total of 4622 cases in 2010 [19].

## 3. Methods

Parametric Hawkes models are conventionally estimated by maximum likelihood estimation (MLE), e.g., [6], and the resulting estimates have desirable asymptotic properties such as consistency, asymptotic normality, and efficiency [20].

In fitting the Hawkes model (1) to the coccidioidomycosis data by MLE, we fit two commonly used different triggering functions, *g*, e.g., [21]. These were the power law function $g\left(u\right)=\left(p-1\right)c^{p-1}{\left(u+c\right)}^{-p}$ and the exponential $g\left(u\right)=\beta e^{-\beta u}$ [22,23,24]. We fit the recursive model (2) with the same exponential triggering form as above and with productivity function $H\left(y\right)=\alpha y^{-q}$.

To evaluate the fit of the Hawkes and recursive models to the data $\left\{\tau_{1},\tau_{2},\ldots ,\tau_{n}\right\}$, observed from Time 0 to time *T*, we used several diagnostics. Since, by the martingale formula $E(\int_{0}^{T}\lambda \left(t\right)dt)=E\left(n\right)$, one way suggested by Harte [25] to check that estimates of the conditional rate λ(t) obtained by MLE are reasonable and not merely local rather than global optima is to inspect the ratio $\frac{\int_{0}^{T}\hat{\lambda }\left(t\right)dt}{n}$, which should be approximately one if the estimated conditional intensity $\hat{\lambda }$ fits well.

Another useful goodness-of-fit diagnostic is the Stoyan–Grabarnik statistic [26], $\sum \frac{1}{T\hat{\lambda }\left(\tau_{i}\right)}.$ Since, as shown in [26],

$$\begin{array}{ccc} E\sum_{i=1}^{n}\frac{1}{\lambda (\tau_{i})} & = & E\int \frac{1}{\lambda (t)}dN.\\ & = & E\int_{0}^{T}1\;dt\\ & = & \;T, \end{array}$$

again by the martingale formula, the Stoyan–Grabarnik statistic should be close to 1 if the model fits well.

A third way to evaluate the fit of point process models is by using superthinning [27]. In superthinning, after choosing some constant b>0, e.g., the total number of observed cases divided by the length *T* of the observation period as suggested in Clements et al. [27], the existing data points are first thinned where each point is randomly kept independently of the others with probability $\min\{\frac{b}{\hat{\lambda }\left(t\right)},1\}$, and then, new points are superposed according to a Poisson process with rate $\max\{b-\hat{\lambda }\left(t\right),0\}$. The resulting superthinned residuals should form a homogeneous Poisson process with rate *b* if and only if $\hat{\lambda }$ is the true conditional rate of the observed point process [16,27]. One may thus inspect the superthinned residuals for uniformity as a diagnostic tool. In particular, if $s_{i}$ are the superthinned times, one may consider the interevent times, $r_{i}=s_{i}-s_{i-1}$ (with the convention $s_{0}=0)$, which should be exponential with mean 1/b if the fitted model $\hat{\lambda }$ is correct, and it is natural therefore to inspect the uniformity of the standardized interevent times $u_{i}=F^{-1}\left(r_{i}\right)$, where *F* is the cumulative distribution function of the exponential with mean 1/b [15]. Clusters, gaps, or other noticeable patterns of nonuniformity in the residuals indicate a lack of fit of the model.

Another tool for comparing the fit of competing models is the Akaike information criterion (AIC) [28], 2p−2L, where *p* is the number of estimated parameters in the model and *L* is the log-likelihood of the model. The preferred model is the one with the minimal AIC, and the difference between nested models is, under the usual regularity conditions, approximately $\chi^{2}$ distributed with p−q degrees of freedom, when comparing models with *p* and *q* parameters, respectively.

In order to guard against overfitting and to investigate forecasting performance, we separated the coccidioidomycosis dataset into two portions, a training set from 1 January 2006 to 28 December 2014, the latter being the last date of records in the year 2014, and a testing portion from 29 December 2014 to 31 December 2017. After re-fitting a model by MLE using only the training data, we inspected its performance during the testing period. For any given week during the testing period, we used the parameters from training and all the data up to the beginning of the week in question and integrated the estimated values of $\hat{\lambda }$ over the week to obtain our forecasted number of events, and assuming an approximately Poisson number of new cases each week, this forecasted rate would also equal the variance of the number of forecast events in the week; this can be used to obtain an approximate 95% confidence interval around each weekly forecast. This forecasted total can be readily compared to the observed number of cases during the week in question.

## 4. Results

**Figure 2:**
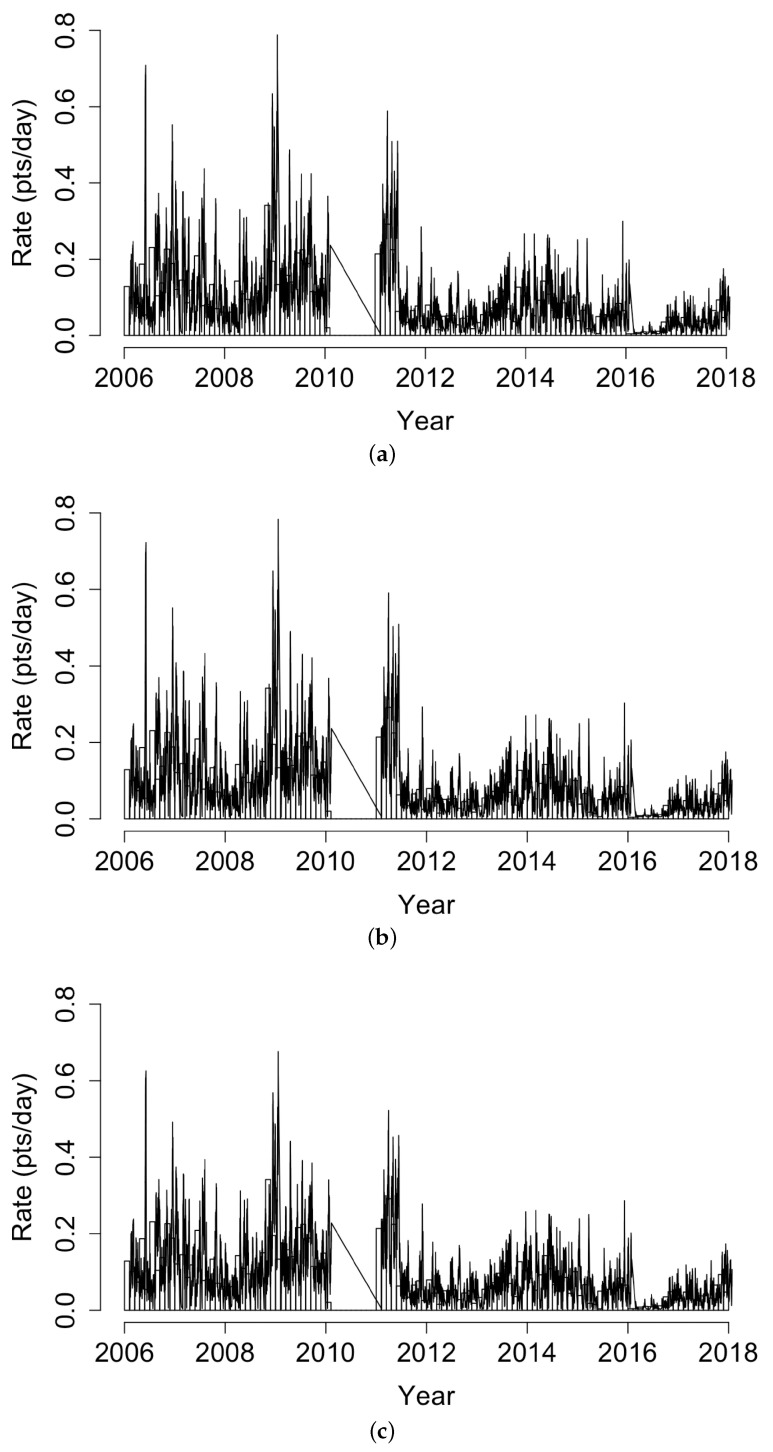
Histogram of coccidioidomycosis cases in California along with the estimated conditional intensity of the fitted Hawkes model with (**a**) power law triggering, (**b**) the Hawkes model with exponential triggering, and (**c**) the recursive model.

The estimated conditional intensities of the fitted Hawkes models with power law or exponential triggering densities and the fitted recursive model are shown in Figure 2, along with the histogram of coccidioidomycosis cases in California. The models were fit by maximum likelihood to the entire coccidioidomycosis dataset; thus, it is not surprising that, retrospectively, the models fit the data closely. The differences in fitted intensities were nearly imperceptible. The mean estimated conditional rate for the three models was 5.55, 5.73, and 5.75 points per day, respectively.

Table 1 shows the martingale ratio $\frac{\int_{0}^{T}\hat{\lambda }\left(t\right)dt}{n}$ and Stoyan–Grabarnik statistic for the recursive and Hawkes models. Neither of these two diagnostics indicated any appreciable sign of a lack of fit for any of the three models. The log-likelihood and AIC are also reported in Table 1 and showed that, for this coccidioidomycosis dataset, compared with the other two models, the recursive model had a significantly higher log-likelihood and lower AIC value, indicating a superior fit to the data.

**Figure 3:**
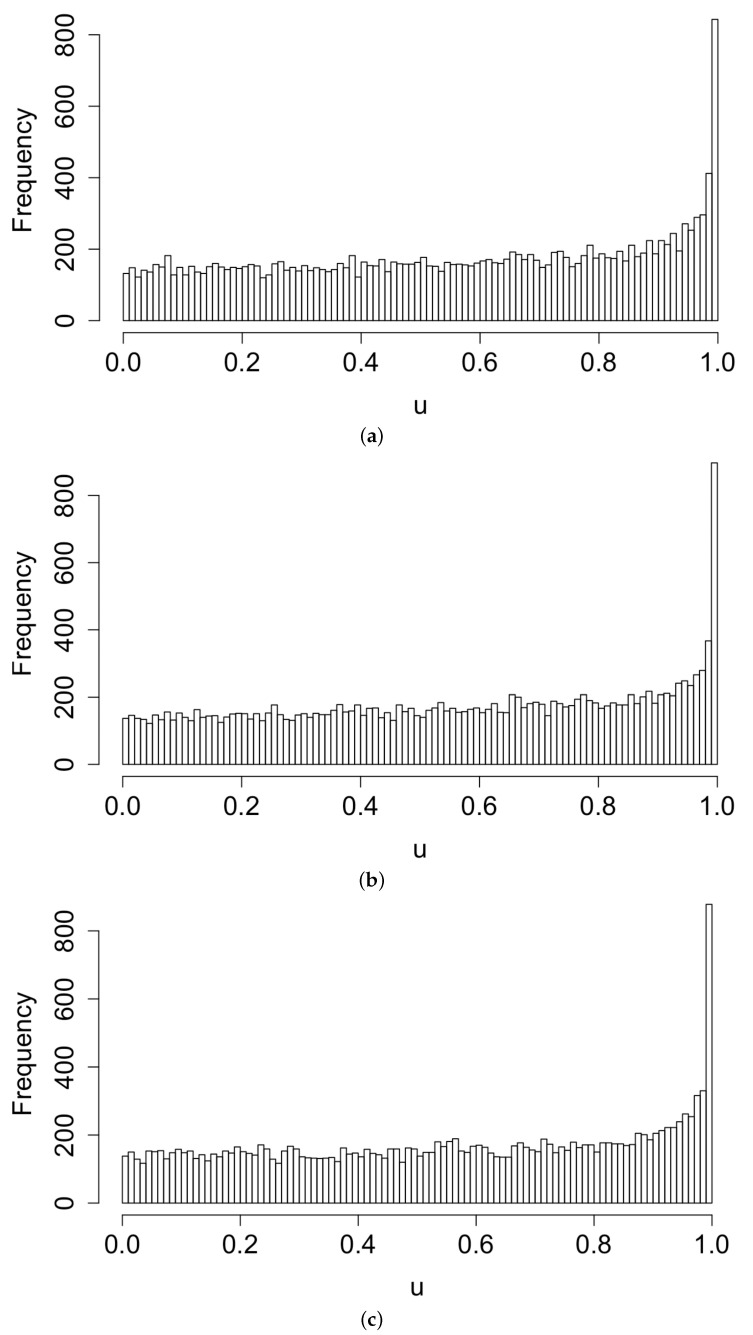
Histogram of standardized interevent times *u* for (**a**) the Hawkes model with power law triggering, (**b**) the Hawkes model with exponential triggering, and (**c**) the recursive model.

**Figure 4:**
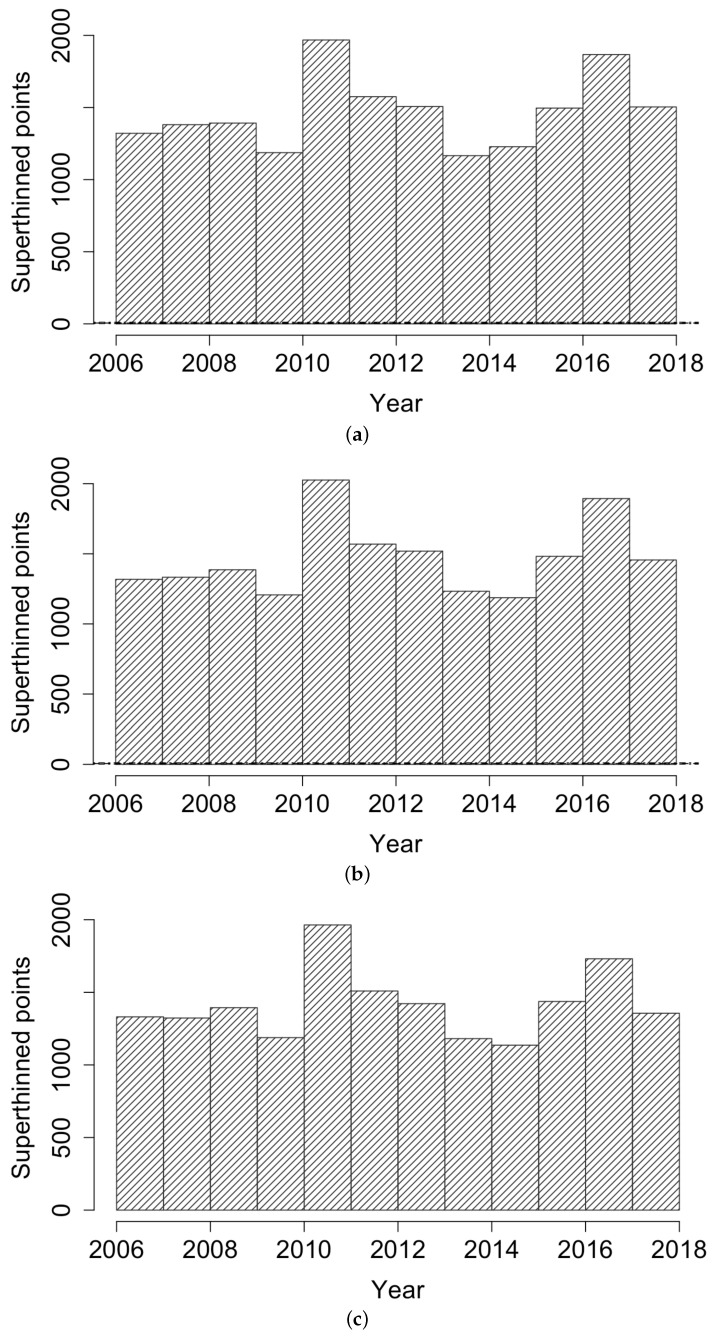
Histograms of superthinned residuals for (**a**) the Hawkes model with power law triggering, (**b**) the Hawkes model with exponential triggering, and (**c**) the recursive model.

**Figure 5:**
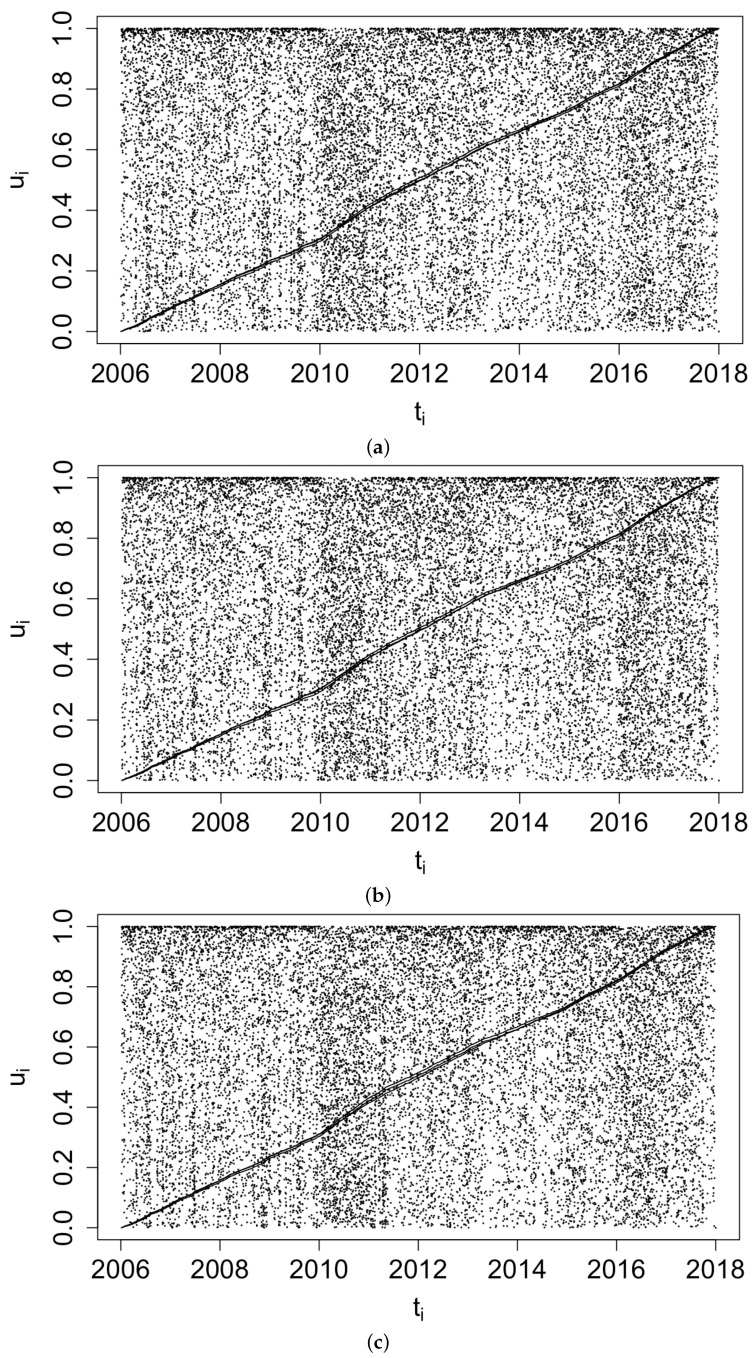
Superthinned residuals $s_{i}$ using b = 100 points/year and their corresponding standardized interevent times $u_{i}$ along with 95% confidence bounds, for (**a**) the Hawkes model with power law triggering, (**b**) the Hawkes model with exponential triggering, and (**c**) the recursive model.

**Figure 6:**
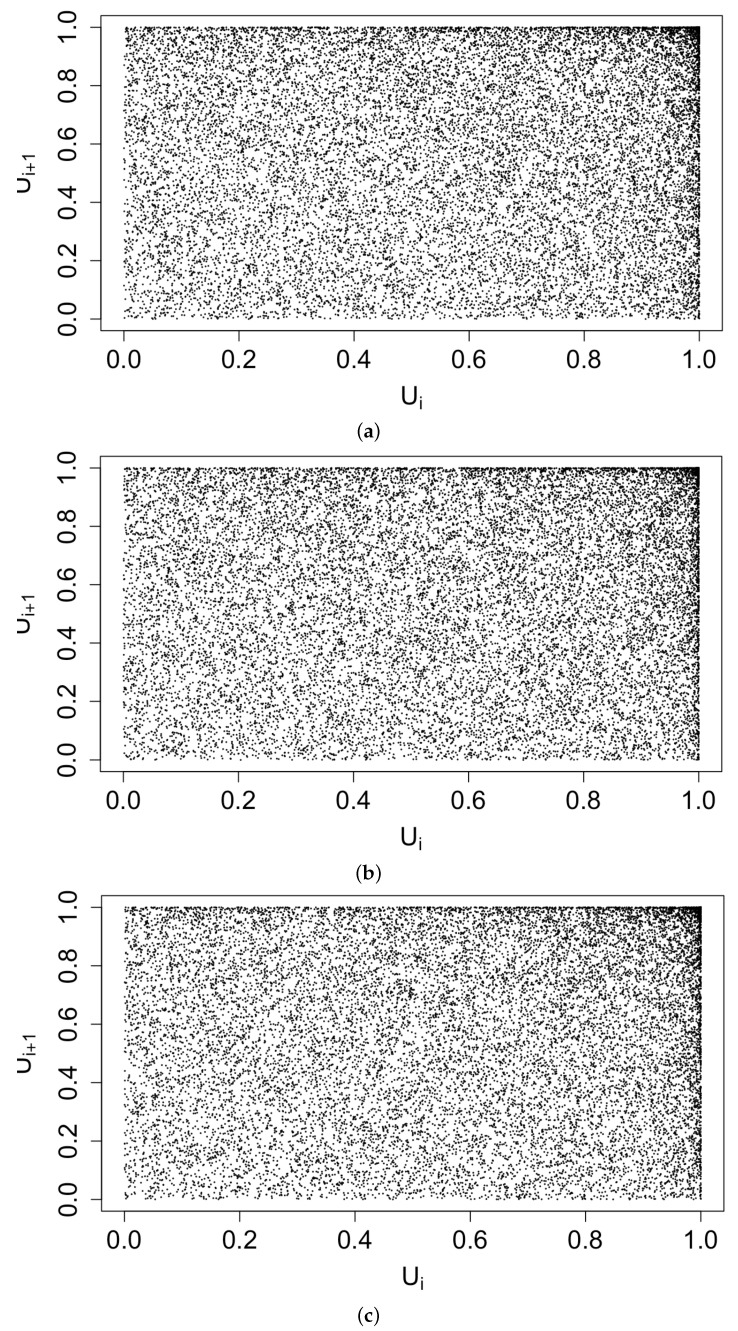
Lag plot of the standardized interevent times $u_{i}$ of the superthinned residuals using b = 100 points/y for (**a**) the Hawkes model with power law triggering, (**b**) the Hawkes model with exponential triggering, and (**c**) the recursive model.

Figure 3 displays histograms of the superthinned points corresponding to each of the three point process models fit to the coccidioidomycosis data. No substantial lack of fit was detected. However, from the standardized interevent times $u_{i}$ for the superthinned residuals shown in Figure 4, one sees that all three interevent time histograms showed some non-uniformity in the form of an excess number of the largest interevent times. This suggests that the models were not adequately accounting for several large gaps in the dataset when no cases were recorded. Figure 5 shows the superthinned residuals $s_{i}$ along with their corresponding standardized interevent times $u_{i}$, as well as the cumulative sum of the standardized interevent times and the individual 95% confidence bounds based on 1000 simulations of an equivalent number of uniform random variables. No clear departures from uniformity were discerned, though the normalized cumulative sum of the standardized interevent times for the Hawkes model with power law triggering exceeded the upper confidence bound from 2008 to 2010 and was lower than the lower bound from the middle of the year 2010 until 2012. Similarly, for the Hawkes model with exponential triggering, the normalized cumulative sum of standardized interevent times was below the lower bound from 2011 to 2013, and similarly for the recursive model from 2010 to 2014. Figure 6 shows lag plots of the standardized interevent times $u_{i}$ of the superthinned residuals for each of the three point process models. Again, no strong evidence of a lack of fit was discerned, other than an excess of large interevent times that were also followed by large interevent times.

**Figure 7:**
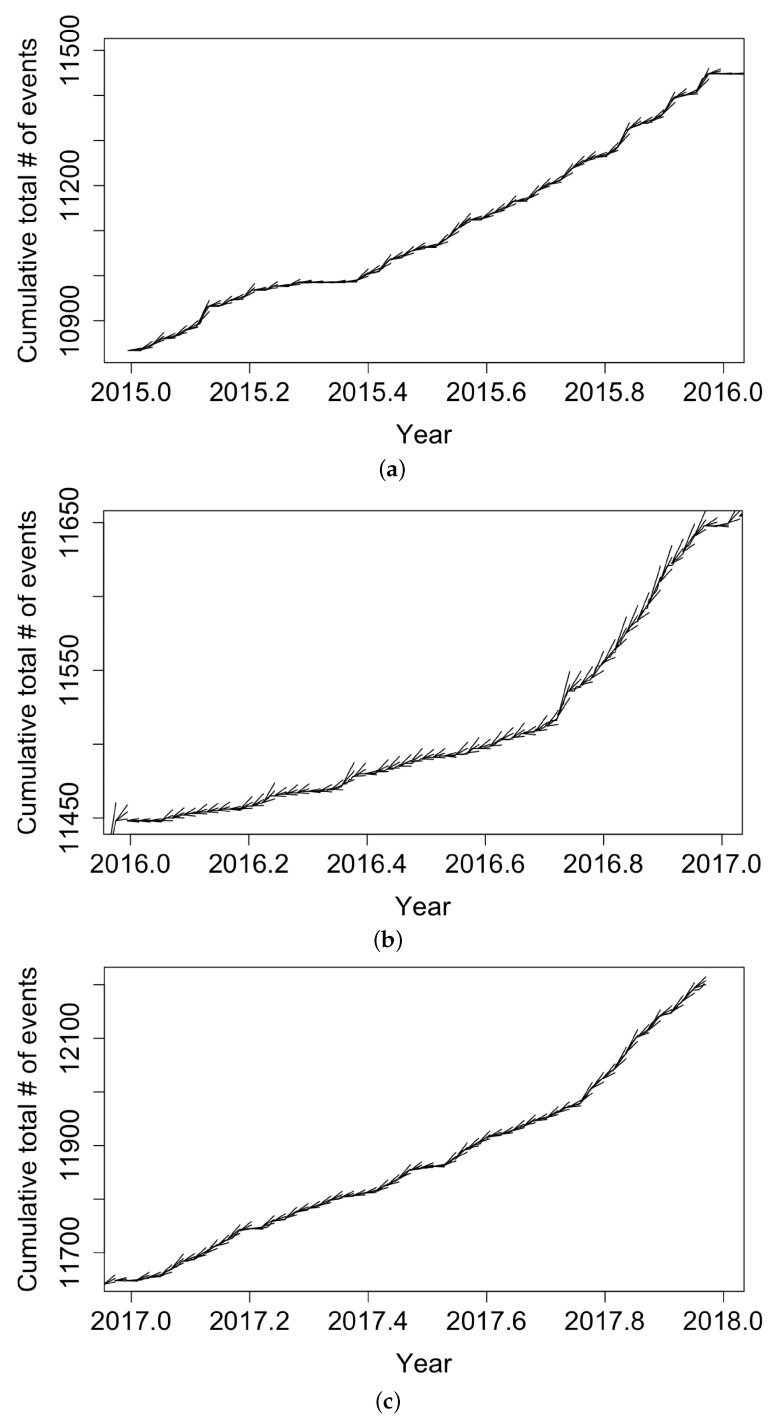
Forecasts of the weekly total number of coccidioidomycosis cases in California using the fitted recursive model and approximate 95% confidence bands, along with reported weekly totals, for (**a**) 2015, (**b**) 2016, and (**c**) 2017. For each week, simulation-based upper and lower 95% confidence bands for the coccidioidomycosis case totals are shown via the whiskers, while the solid black curve shows the observed case count totals.

To guard against overfitting and to see how the recursive model would perform in forecasting, we re-fit the recursive model using only training data from 1 January 2006 to 28 December 2014, yielding parameter estimates $(\hat{\mu }=0.0507$ pts/day, $\hat{\alpha }=1.284$ pts/day, $\hat{q}=0.1737$, $\hat{\beta }=0.6501)$ points/day, with estimated standard errors of 0.01459, 0.04004, 0.01825, and 0.02561, respectively. The fitted value of $\hat{\mu }$ indicates that, according to the fitted model, only approximately 18.5 cases per year were attributable to the background or immigration of cases, with the remainder of points attributed to triggering, and the estimated mean triggering time in the fitted model was $1/\hat{\beta }=1.54$ days. The positivity of $\hat{q}$ indicates higher productivity when the conditional intensity was low, in agreement with the hypothesis that transmission rates are highest at the beginning of the outbreak. Figure 7 displays the forecasting results from the fitted recursive model. Some forecasts exceeded the observed number of events, especially at the end of 2015–2016. However, the recursive model seemed to perform well in the testing period, with most realizations falling within two standard errors from our predictions. The root mean squared error for the recursive model forecasts was 3.62 events/day.

## 5. Discussion

Coccidioidomycosis is an infectious disease of mammals, including humans, caused by inhaling spores of one of two species of fungi, that rarely spreads between hosts [29]. Nevertheless, the results here suggest that the Hawkes and recursive point process models, previously successfully applied to contagious diseases that move between hosts (e.g., Ebola), were also able to capture the main features of diagnosed coccidioidomycosis cases in California. Indeed, the clustering apparent in the California coccidioidomycosis data appeared to be well summarized by the point process models, particularly the recursive model. It is important to note that clustering attributed by the model to *causal* triggering from one individual to another may more likely be attributable to external factors, such as fungal density, spore activity, or disturbance dynamics, or changes to community exposure or environmental conditions [30], which were not recorded as part of this dataset. In all aspects of the fitting and evaluation of the spread of the infection we performed, the recursive point process model appeared to fit slightly better than the corresponding Hawkes point process models with power law or exponential triggering functions. However, future work should focus on more complex models where fungal (spore and adult) and human population densities, as well as exposure likelihood are explicitly modeled. We hope this work will spur the collection of such data in the future, as well as draw attention to the limitations of the coccidioidomycosis data considered here, especially unreported cases and missing data issues that may have biased the present analysis. Despite these caveats, the ability of point process models to accurately capture incidence increases in emergent infectious diseases, regardless of the means of transmission, presents an exciting new opportunity to inform public health and mitigation efforts during future outbreak events.

## Figures and Tables

**Table 1:** Test Result Comparison Table.

	Hawkes Power Law	Hawkes Exponential	Recursive
Martingale Ratio	1.0000254	0.999974	1.002272
Stoyan–Grabarnik	0.9996276	0.999956	1.000336
Log-Likelihood	5499.55	5498.71	5525.49
AIC	−10,991.11	−10,991.42	−11,042.99

## Data Availability

Coccidioidomycosis data were provided by Project Tycho, https://www.tycho.pitt.edu (accessed on 7 May 2021), and were compilations of published reports by the World Health Organization and the Centers for Disease Control and Prevention.
